# Ultrasound enhanced siRNA delivery using cationic liposome-microbubble complexes for the treatment of squamous cell carcinoma

**DOI:** 10.7150/ntno.90516

**Published:** 2024-03-09

**Authors:** Bin Qin, Xucai Chen, Jianhui Zhu, Jonathan Kopechek, Brandon Helfield, Francois Yu, Jissy Cyriac, Linda Lavery, Jennifer R. Grandis, Flordeliza S. Villanueva

**Affiliations:** 1 Center for Ultrasound for Molecular Imaging and Therapeutics, University of Pittsburgh Medical Center, University of Pittsburgh, Pittsburgh, PA, USA.; 2 Department of Otolaryngology-Head and Neck Surgery, University of California, San Francisco, CA, USA.

**Keywords:** Ultrasound Targeted Microbubble Cavitation, Squamous Cell Carcinoma, Tumor Growth Inhibition, Head and Neck Cancer

## Abstract

**Rationale:** Microbubble (**MB**) contrast agents combined with ultrasound targeted microbubble cavitation (**UTMC**) are a promising platform for site-specific therapeutic oligonucleotide delivery. We investigated UTMC-mediated delivery of siRNA directed against epidermal growth factor receptor (**EGFR**), to squamous cell carcinoma (**SCC**) via a novel MB-liposome complex (**LPX**).

**Methods: LPXs** were constructed by conjugation of cationic liposomes to the surface of C_4_F_10_ gas-filled lipid MBs using biotin/avidin chemistry, then loaded with siRNA via electrostatic interaction. Luciferase-expressing SCC-VII cells (**SCC-VII-Luc**) were cultured in Petri dishes. The Petri dishes were filled with media in which LPXs loaded with siRNA against firefly luciferase (**Luc siRNA**) were suspended. Ultrasound (**US**) (1 MHz, 100-µs pulse, 10% duty cycle) was delivered to the dishes for 10 sec at varying acoustic pressures and luciferase assay was performed 24 hr later. *In vivo* siRNA delivery was studied in SCC-VII tumor-bearing mice intravenously infused with a 0.5 mL saline suspension of EGFR siRNA LPX (7×10^8^ LPX, ~30 µg siRNA) for 20 min during concurrent US (1 MHz, 0.5 MPa spatial peak temporal peak negative pressure, five 100-µs pulses every 1 ms; each pulse train repeated every 2 sec to allow reperfusion of LPX into the tumor). Mice were sacrificed 2 days post treatment and tumor EGFR expression was measured (Western blot). Other mice (*n*=23) received either EGFR siRNA-loaded LPX + UTMC or negative control (**NC**) siRNA-loaded LPX + UTMC on days 0 and 3, or no treatment (“sham”). Tumor volume was serially measured by high-resolution 3D US imaging.

**Results:** Luc siRNA LPX + UTMC caused significant luciferase knockdown vs. no treatment control, *p*<0.05) in SCC-VII-Luc cells at acoustic pressures 0.25 MPa to 0.9 MPa, while no significant silencing effect was seen at lower pressure (0.125 MPa). *In vivo,* EGFR siRNA LPX + UTMC reduced tumor EGFR expression by ~30% and significantly inhibited tumor growth by day 9 (~40% decrease in tumor volume vs. NC siRNA LPX + UTMC, *p*<0.05).

**Conclusions:** Luc siRNA LPXs + UTMC achieved functional delivery of Luc siRNA to SCC-VII-Luc cells *in vitro*. EGFR siRNA LPX + UTMC inhibited tumor growth and suppressed EGFR expression *in vivo*, suggesting that this platform holds promise for non-invasive, image-guided targeted delivery of therapeutic siRNA for cancer treatment.

## Introduction

Small interfering RNA (**siRNA**) as a therapeutic offers great promise for many incurable and difficult to treat diseases, due to its high specificity and capability of potent gene silencing 1-3. In general, naked siRNA is not readily taken up by cells via passive diffusion due to its relatively large molecular weight and negative charge. These unfavorable characteristics, along with inherent physiological barriers, make systemic siRNA delivery very challenging 4.

Ultrasound-targeted microbubble cavitation (**UTMC**) is an emerging delivery approach for local, site-specific delivery of a therapeutic payload (e.g. a drug or genetic material). Microbubbles (**MBs**), employed as an ultrasound (**US**) contrast agent, consist of a suspension of micron-sized (1-10 µm), gas-filled microspheres stabilized by a biocompatible shell 5. Due to the large compressibility of the gaseous core, MBs oscillate in an US pressure field and are strong scatterers of US. At low driving amplitudes, microbubbles exhibit “stable” cavitation, defined as a repeated vibration about its equilibrium size. This can develop into small-scale acoustic streaming and the generation of local sustained shear stresses. At higher driving amplitudes, MBs can be characterized by “inertial” cavitation, which may lead to violent behaviors including MB disruption and microjet formation. Both these cavitation regimes may lead to a transient perforation of the plasma membrane of nearby cells 6. This process, known as sonoporation, has been utilized to promote the intracellular uptake and transport of genetic material and/or therapeutic drug payloads, or to permit delivery of cargos into extravascular space of surrounding tissues *in vivo* 7-14.

UTMC has been explored broadly to deliver genes *in vitro* and *in vivo*. Early reports have shown that co-administration of naked siRNA and neutral MBs can deliver siRNA into cells through sonoporation, however, at very low delivery efficiencies 15. Another approach is to employ cationic lipid MBs to carry oligonucleotides on the MB shell via electrostatic interaction. Upon MB disruption, the release of a large, local concentration of oligonucleotides may result in oligonucleotide diffusion into cells through transient pores 16. Compared with the co-administration method, conjugating oligonucleotides to cationic MBs is usually more effective 17, 18, and it prevents the dilution of oligonucleotides after they enter the blood circulation.

*In vivo* studies from our and other groups revealed that cationic lipid MBs can deliver therapeutic siRNA or plasmid into the target tissue and trigger biological activity 16, 19, 20. One issue associated with cationic MBs, however, is the limited oligonucleotide-carrying capacity. Administration of a large amount of MBs is usually required to fulfill the required dose of oligonucleotide for *in vivo* study. For instance, in a previous preclinical study using this technique for gene delivery, the amount of cationic lipid MBs required was on the order of 10-100 times the typical human dosage of MBs 21, underscoring the need to improve the ability to deliver more oligonucleotide for a given dose of MBs.

The most frequent approach to loading drugs on to MBs for MB-triggered drug delivery has involved placement of the payload on the outside surface of the thin microbubble shell, which inherently limits loading capacity. Efforts to improve MB loading or increase drug delivery have been reported 22. For example, modulation of lipid microbubble DNA loading capacity was increased by addition of steric acid modified polyethyleneimine 600 (Stearic-PEI600) to the shell, but was associated with cytotoxicity 23. Loading of drugs on to the surface of albumin microbubbles can be increased by chemical modifications; e.g., disulfide bonds and glutaraldehyde cross-linking 24. Lipid shell microbubbles with longer lipid chains have longer *in vivo* half-life 25 and potentially higher delivery efficiency for larger molecules, but optimal acoustic conditions for delivery are not well-defined 26. A formulation of 'antibubbles,” comprising liquid droplets surrounded by a gas layer has been described, may allow large volumes of payload to be carried by the internal droplets, but is in very early proof of concept testing stages 27. Drug-loaded nanoparticles linked to the microbubble shell have been described 28, 29. However, to date, studies of *in vivo* efficacy of such constructs for functional delivery of oligonucleotides are lacking. As such, novel MB design with a view to improving the nucleic acid loading capacity while maintaining functional delivery of the payload is critical to translating US-stimulated MB oligonucleotide delivery in the clinical setting. To this end, we designed a microbubble-liposomal complex that combines the high loading capacity of liposomes with the targeted delivery capabilities of microbubble cavitation in an ultrasound field.

Therefore, novel MB design with a view to improving the nucleic acid loading capacity is critical to translating US-stimulated MB oligonucleotide delivery approaches to the clinical setting.

The objective of this study was to develop and characterize a novel microbubble-liposome complex to enhance the localized delivery of siRNA. The microbubble-liposome formulation was first characterized in terms of its siRNA loading capacity, and then assessed *in vitro* over a range of US exposure conditions for its ability to cause sonoporation and deliver siRNA, as measured by luciferase knockdown in a luciferase reporter cell line. Finally, *in vivo* US-mediated delivery of siRNA against epidermal growth factor receptor (**EGFR**) was investigated in a murine model of squamous cell carcinoma (**SCC**).

## Methods

### Chemicals and cell culture

The siRNA targeted to GL4 luciferase (Sense strand: 5'- GGACGAGGACGAGC-ACUUCUU-3', Antisense strand: 3'-UUCCUGCUCCUGCUCGUGAAG-5') was custom synthesized (Thermo Fisher Scientific, Carlsbad, CA). Negative control (**NC**) siRNA (Silencer^®^ Negative Control No. 1 siRNA) and the siRNA targeted to mouse EGFR gene (silencer® s65374, EGFR siRNA) were ordered from Thermo Fisher Scientific. Murine squamous cell carcinoma (SCC-VII) cells were maintained in RPMI-1640 medium supplemented with 10% fetal bovine serum (**FBS**) and 2% penicillin/streptomycin. SCC-VII cells stably expressing luciferase (**SCC-VII-Luc**) were generated by transfecting SCC-VII cells with Cignal Lenti signal transducer and transcription activator 3 (STAT3) Reporter (luc) Kit (Qiagen, Valencia, CA) which contains a VSV-g pseudo typed lentivirus particle expressing the firefly luciferase gene under the control of a CMV promoter and the tandem repeats of STAT3 transcriptional response element. The lentivirus transfected SCC-VII cells were selected with 0.75 µg/mL puromycin (MP biomedicals, Santa Ana, CA) and maintained in RPMI-1640 medium supplemented with 10% FBS, 2% penicillin/streptomycin and 0.75 µg/mL puromycin. Cells were cultured at 37 °C in a humidified atmosphere containing 5% CO_2_. All cell culture products were obtained from Lonza (Allendale, NJ).

### Lipid MB preparations

Lipid MBs were prepared from a lipid aqueous dispersion composed of polyoxyethylene(40) stearate (Sigma-Aldrich; St. Louis, MO), 1,2-distearoyl-sn-glycero-3-phosphocholine (DSPC), and 1,2-distearoyl-sn-glycero-3-phosphoethanolamine-N-[bio-tinyl(polyethylene glycol)-2000] (DSPE-PEG2000-biotin) (Avanti polar lipids; Alabaster, AL), as previous described 30. Briefly, polyoxyethylene (40) stearate, DSPC and DSPE-PEG2000-biotin (1/2/1, w/w/w) was dissolved in chloroform. The chloroform was evaporated by flushing with argon, followed by overnight vacuum-drying. The dried lipid film was rehydrated in 0.9% sodium chloride saline (final lipid concentration as 10 mg/mL) for 4 hr at room temperature. After a brief tip sonication to dissolve any lipid debris, the lipid dispersion was sonicated with a 20 kHz probe (Heat Systems Ultrasonics, Newtown, CT) in the presence of perfluorobutane gas (FluoroMed, L.P., Round Rock, TX). After sonication, the MBs were washed with 20 mL saline twice to remove any free lipid and were suspended in saline saturated with perfluorobutane. The lipid MBs were aliquoted in vials with perfluorobutane filled in head space and were stored at 4 ºC until use.

### Cationic liposome preparation

DOTAP, DOPE and DSPE-PEG2000-biotin were individually dissolved in chloroform and mixed with molar ratio of 49:49:2. The chloroform was evaporated by flushing with argon and followed by overnight vacuum-drying. The dried lipid film was rehydrated in deionized water for 30 min at 65 ºC. The crude liposomes were extruded through two stacked of 400 nm polycarbonate membrane for 10 times at 65 ºC. The free lipids were removed by passing through a Sephadex G-50 column (GE Health Care). The cationic liposome was stored in 4 ºC until use.

### Conjugation of cationic liposome with lipid MB

**Figure 1:**
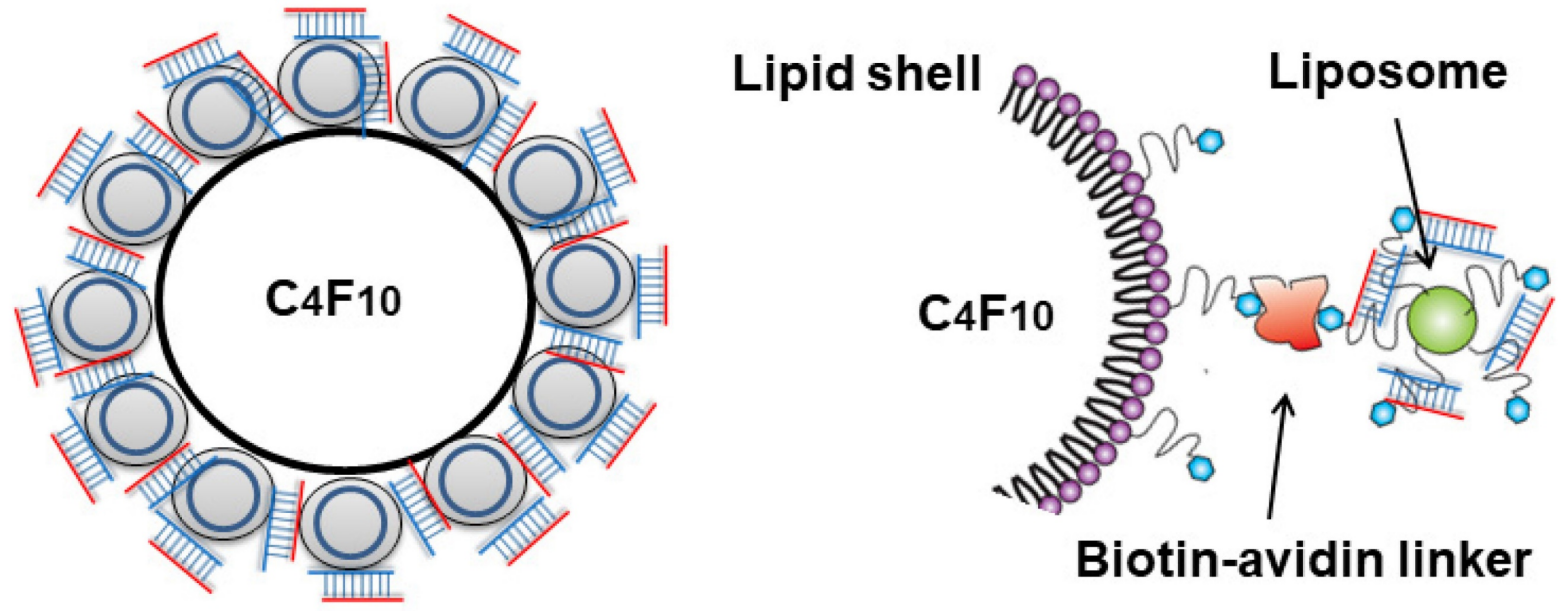
Schematic diagram of LPX.

The cationic liposomes were conjugated to lipid MB via biotin-streptavidin interaction (**Figure 1**). The streptavidinylated MB suspension was prepared by incubating 2×10^8^ MBs with 0.83 mg/mL streptavidin (Thermo Fisher Scientific). After 30 min of incubation, the MBs were washed twice with 1 mL PBS, by 3 min centrifugation at 200 g to remove free streptavidin. The resulting streptavidinylated MB suspension was slowly added to saline containing excess amounts of cationic liposomes. After 30 min incubation with constant agitation, the MBs were washed twice with 1 mL saline by 3 min centrifugation at 200 g to remove unbound liposomes. The liposome-MB conjugates (lipoplex, **LPX)** were further incubated with various amounts of siRNA (2-20 µg) for 5 min to allow siRNA loading, then washed twice with 1 mL PBS.

### Liposome and MB characterization

The concentration and size distribution of MBs were measured by a Multisizer-3 coulter counter (Beckman Coulter, Brea, CA). The size and zeta potential of the cationic liposomes were measured using a Zetasizer-Nano ZS (Malvern instruments, Worcestershire, UK).

### Quantification of siRNA loading to LPX

To quantify the siRNA loading affinity of the LPX, 1×10^8^ LPXs were incubated with serial doses of EGFR siRNA (2, 5, 10, 15, 20 µg) in 0.9% saline for 20 min at room temperature. The samples were centrifuged at 200g for 3 min to separate LPX from unbound siRNA. Both subnatant and supernatant (containing the LPX) were harvested separately. After 20 min of incubation, the MBs were washed twice with PBS. The siRNA bound to LPX was released by incubation with heparin for 10 min and analyzed by agarose gel electrophoresis with Gel-Red (Biotium, Fremont, CA) staining.

### Protocol for siRNA Delivery with UTMC *in vitro*

**Figure 2:**
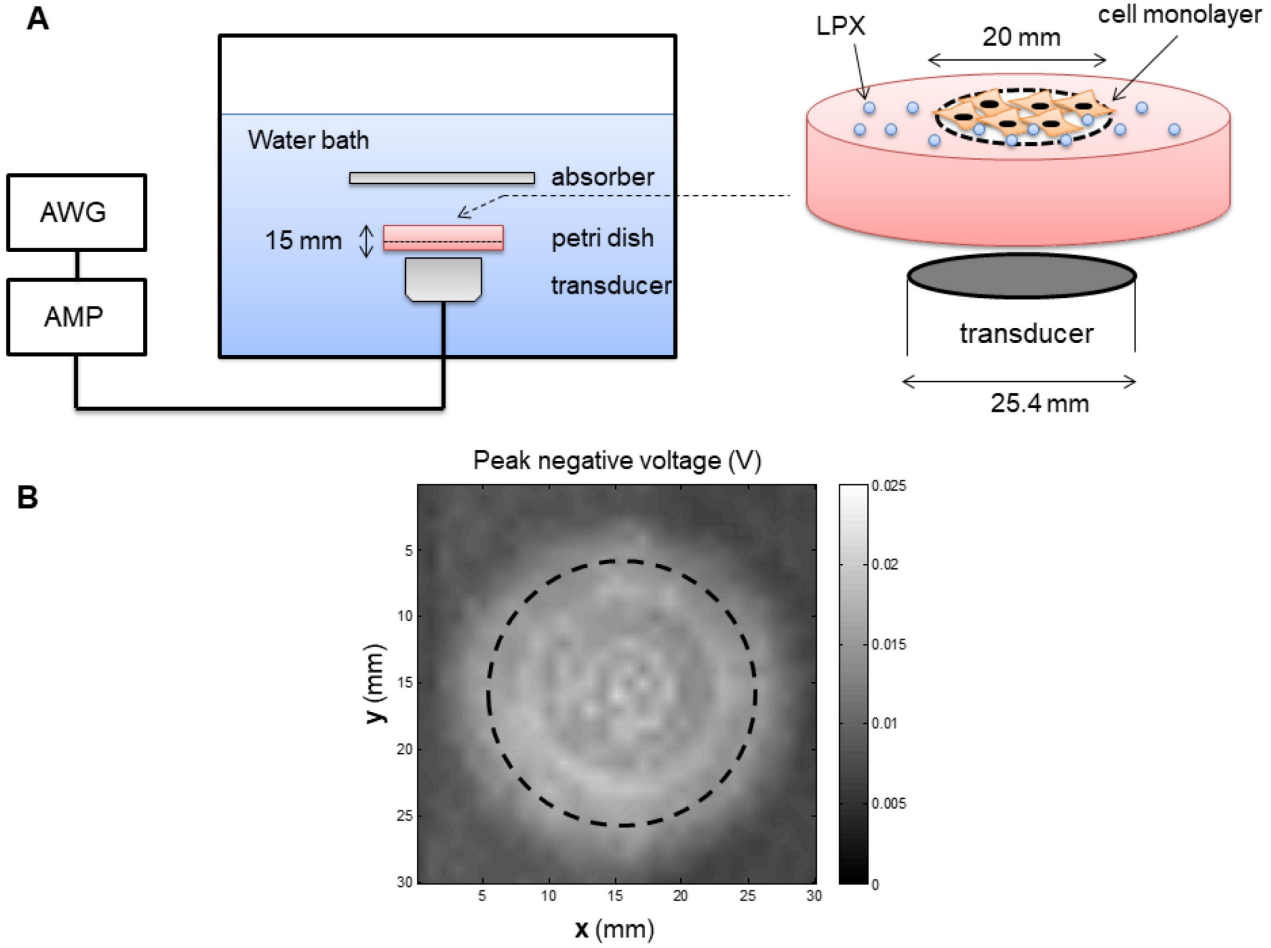
(**A**) Experiment setup of *in vitro* studies. AWG: arbitrary function generator; AMP: Amplifier. (**B**) Map of the acoustic field of the 25.4 mm transducer used for *in vitro* experiment, measured in the absence of the *in vitro* setup in a separate water tank. Dashed line indicates the footprint of the well.

An inverted dish setup was used to test the siRNA delivery capability of LPX *in vitro* (**Figure 2A**). A sterilized plastic ring (20 mm diameter) was affixed on the center of a 45 mm-diameter petri dish (Pall Corporation) using petroleum jelly. Before cell culturing, the circular area inside the ring was pre-coated with fibronectin (0.25 µg/cm^2^) to enhance cell attachment. SCC-VII-Luc cells (1×10^5^ cells) were seeded inside the ring. After overnight incubation, the medium and the ring were removed, and the petri dish was fully refilled with medium and sealed with a cap. The MBs were injected into the petri dishes through a hole on the cap. Before UTMC, the petri dishes were inverted for 5 min to allow luciferase siRNA-loaded LPX (**Lux siRNA LPX**) to float to close proximity to the cell monolayer. The inverted dish was submerged within a degassed water tank maintained at 37ºC. US was generated by a 1 MHz, 25.4 mm diameter flat single element transducer (A302S-SU, Olympus America Inc, Waltham, MA) located directly underneath the petri dish, excited by an arbitrary function generator (AFG3252, Tektronics, Beaverton, OR) and a power amplifier (250A250AM8, Amplifier Research, Souderton, PA). The US field was calibrated using a hydrophone (HGL-0200, Onda Corp, Sunnyvale, CA) in a separate water tank (**Figure 2B**), at the level of the cell monolayer, 15 mm from the transducer surface. A range of acoustic pressures (1 MHz, 0.125 MPa to 0.9 MPa spatial peak temporal peak negative pressure, 100 µs pulse, pulse interval 1 ms, 10 sec exposure time) were tested.

### Measurement of UTMC-mediated sonoporation and Luc-siRNA delivery *in vitro*

Propidium iodine (PI) (Sigma Aldrich, St. Louis, MO) is a membrane impermeable dye that is usually excluded from cells with intact cell membranes. SCC-VII-Luc cells mixed with Luc siRNA LPX, PI (0.21 mg/mL), and the membrane-permeable dye Hoechst 33342 (2 µg/mL), were insonified using the inverted dish setup and US parameters described above. Ten minutes after UTMC, the cells were washed with PBS and the cell viability was assessed using calcein-AM (2 µM), a membrane-permeable nonfluorescent compound that converts into fluorescent calcein after hydroxylation by intercellular esterase. Cell samples were then examined with an inverted microscope (IX81, Olympus, Center Valley, PA), interfaced with a digital charge-coupled device camera (DP71, Olympus). Digital images were processed using MATLAB (MathWorks, Natick, MA) offline. Cells with positive PI and calcein staining were defined as sonoporated cells, and the percentage of sonoporated cells over the total number of cells (as measured from the Hoechst staining) was defined as sonoporation efficiency. Dead cells usually have no calcein signal or very week spotty calcein signal.

In separate *in vitro* UTMC studies using the protocol described above, twenty-four hours after UTMC treatment, cells were lysed with a reporter lysis buffer (Promega, Madison, WI), and the luciferase assay was performed using a luciferase assay system (Promega, Madison, WI) on a tube luminometer (Lumat^3^ LB9501, Berthold Technologies, Bad Wildbad, Germany) using the protocol provided by the manufacturer. The relative luciferase activity was calculated by normalizing the results with the total protein concentration, which was quantified using Bradford Assay kit (ThermoFisher, Pittsburgh, PA).

### siRNA Delivery with EGFR siRNA LPX + UTMC to murine SCC *in vivo*

We next sought to test our siRNA LPX + UTMC delivery platform in an *in vivo* murine SCC model. All animal procedures were approved by the University of Pittsburgh Institutional Animal Care and Use Committee. A SCC tumor model was generated by subcutaneous injection of 1.5×10^6^ SCC-VII cells into immunocompetent C3H mice as previously described 10, 19. Mice were anesthetized using isoflurane and a catheter was placed in the internal jugular vein for MB infusion. A 0.5 mL volume of LPX suspension in saline, containing 7×10^8^ LPX loaded with ~30 µg of epidermal growth factor receptor (**EGFR**) siRNA (**EGFR siRNA LPX**) or negative control (**NC**) siRNA (**NC siRNA LPX** ) was infused for 20 minutes using a syringe pump set at 1.5 mL/h. Concurrent US (1 MHz, 0.5 MPa spatial peak temporal peak negative pressure, 100-µs pulse repeat 5 times every 1 ms, with the complex pulse train repeated every 2 sec to allow reperfusion of MB into the treatment area, overall duty cycle 0.025%) was delivered over 25 minutes to the tumor using a 12.7 mm diameter flat disk transducer (A303S-SU, Olympus NDT) mounted at fixed position. The treatment was monitored by imaging the tumor using a 15L8 transducer probe on a Sequoia 512 US imaging system (Siemens Ultrasound, Issaquah, WA) operated in Contrast Pulse Sequencing mode (7 MHz, mechanical index=0.2, and frame rate of 10 Hz) to confirm MB destruction by UTMC and reperfusion of MBs in the tumor. Additionally, MB behavior in the tumor was recorded with a passive cavitation detector in a subset of animals during LPX + UTMC treatment. For this purpose, a broad band focused single element transducer with a center frequency of 3.5 MHz (V383, 9.5 mm diameter, 25.5 mm focus, -6 dB beam diameter 1.2 mm, Olympus NDT) was co-aligned with the treatment transducer for the detection of MB cavitation events. The detected radio-frequency signal was amplified, digitized, and power spectrum as analyzed. The presence of ultra-harmonics would indicate stable cavitation and broadband signal would indicate the presence of inertial cavitation.

For tumor growth inhibition studies (*n*=7-8 mice per group), treatment was started when tumor volumes were between 20-50 mm^3^, usually 5 to 6 days after SCC-VII cell inoculation. The animals received a total of two EGFR siRNA LPX + UTMC treatments on days 0 and 3, and the tumor volume was quantified every 3 days using a high-resolution 3D US imaging system (Vevo 2100, Visualsonics, Ontario, Canada). For comparison, a group of animals received NC siRNA LPX + UTMC treatments on days 0 and 3 under otherwise identical conditions. A separate group of tumor-bearing mice underwent identical surgical venous line placement and anesthesia on days 0 and 3, but received only i.v. saline (sham control). All animals were euthanized under deep anesthesia (5% isoflurane) on day 9 after the last volume measurement. To assess the EGFR silencing effect, mice were sacrificed 2 days after receiving one treatment and tumor EGFR expression was measured by Western blot and immunofluorescence.

### Western blot

Tumors were homogenized in ice cold RIPA buffer (150 mM sodium chloride, 1% Triton X-100, 0.5% sodium deoxycholate, 0.1% sodium dodecyl sulfate, 50 mM Tris, pH 8.0) supplemented with proteinase inhibitor cocktail (Roche Applied Science, Penzberg, Germany). Tissue lysates were then electrophoresed and transferred to PVDF membranes (Millipore, Billerica, MA). The membranes were blocked and incubated with anti-EGFR antibody (1:1000 dilutions) or anti-β-actin antibody (1:1000 dilution). The membrane was then washed and incubated with horseradish peroxidase conjugated secondary antibody, washed, and developed with ECL (Thermo Fisher Scientific, Rockford, IL). Images were captured on X-ray film.

### Histology and immunofluorescence assessment

Frozen tumor tissues were sectioned in 10 µm thickness using a Leica CM3050 S cryo-microtome (Leica Biosystems Ltd., Newcastle) and fixed in ice-cold methanol. The sections were stained with haematoxylin and eosin and examined using an Olympus IX81 microscope interfaced with a cooled EM-CCD camera (C9200-2, Hamamatsu Photonics KK, Hamamatsu, Japan). For immunofluorescence assessment, frozen sections were fixed in cold methanol for 20 min. After PBST washing, sections were blocked in normal goat serum for 30 min and then incubated with rabbit anti-EGFR (1:100, Santa Cruz Biotechnology, Inc, Dallas, TX) overnight at 4 ºC, followed by Goat anti-rabbit Alexa Fluo^®^488 conjugated Goat anti-Rabbit antibody (1:500, Molecular Probes, Thermo Fisher Scientific). For negative control, EGFR antibody was replaced with normal rabbit IgG.

### Statistical analysis

Data were expressed as the mean ± standard deviation (SD). Difference among groups was determined using one-way ANOVA. When a significant difference was found (*p*<0.05), *post hoc* testing using Student's *t*-test was performed to determine where the difference resided, with *p*<0.05 considered statistically significant.

## Results

### Characterization of LPX

**Figure 3:**
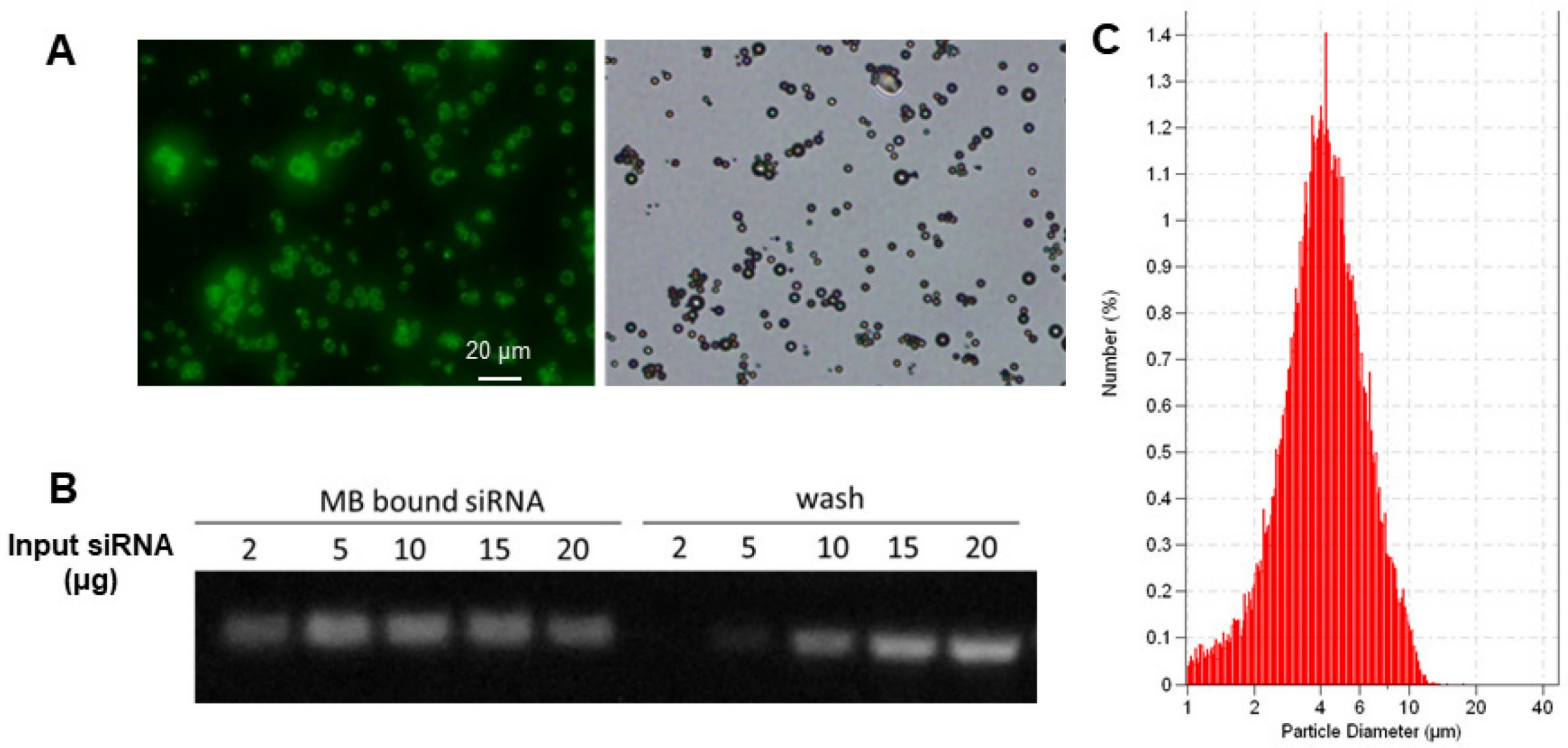
(**A**) Fluorescence (left) and brightfield (right) microscopic images of lipid MBs conjugated with FITC labelled liposomes via biotin/streptavidin chemistry; (**B**) EGFR siRNA loading capacity of LPX determined by gel electrophoresis. 1×10^8^ of LPX was saturated by about 5 µg siRNA. (**C**) Particle size histogram of LPX.

The biotinylated cationic liposomes exhibited a typical size distribution with a mean diameter of 152±21 nm, and a zeta potential of 46.2±2.1 mV. To confirm that cationic liposomes can be conjugated to MB, fluorescein-labeled biotinylated cationic liposomes were incubated with streptavidin coated MB. Fluorescence microscopy observation showed there was intense and uniform fluorescence signal on the lipid MBs (**Figure 3A**), indicating successful attachment of liposomes on the MB shell. The siRNA binding affinity was quantified by incubation of LPX with increasing amounts of EGFR siRNA. The LPX was found to be capable of loading ~5 µg siRNA per 1×10^8^ MB (**Figure 3B**). After loading with siRNA, the final construct has a mean diameter of ~ 4 µm (**Figure 3C**), as measured with a Coulter counter. Since the LPX was saturated with siRNA, it was negatively charged, with a zeta potential of ‑(16±2.1) mV.

### Sonoporation efficiency of LPX + UTMC

**Figure 4:**
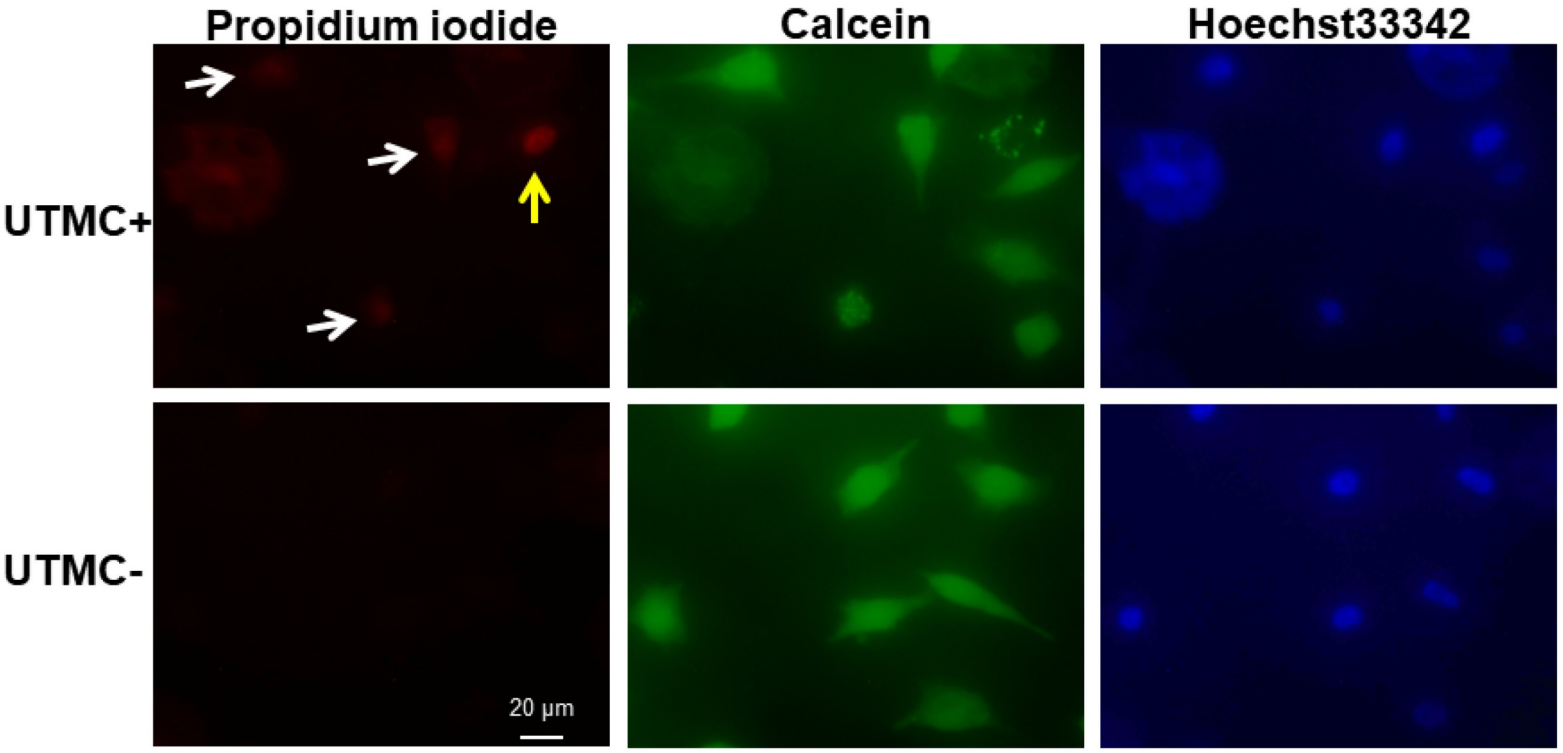
After LPX + UTMC treatment, cell membrane poration was assessed using PI and cell viability was determined using calcein-AM. Sonoporated cells are cells positively stained with both PI and calcein (white arrow). Dead cells are PI positive but calcein negative (yellow arrow). For this example, acoustic pressure was 0.25 MPa.

**Figure 5:**
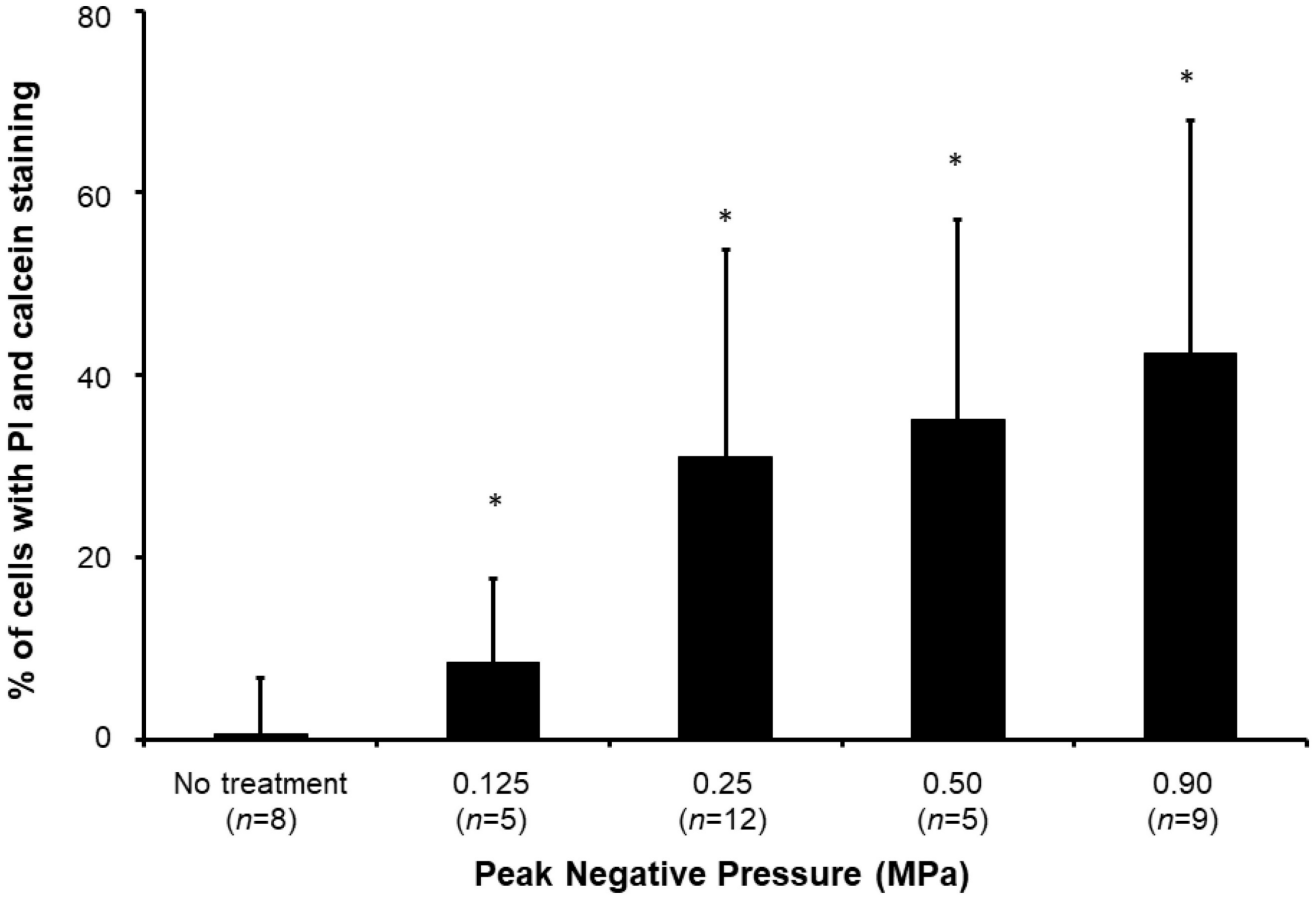
Sonoporation efficiency of LPX + UTMC under various acoustic pressures. (*n*=5-12). *p<0.05 vs. no treatment control.

Sonoporated cells typically had moderate PI signal and strong and diffusive calcein staining in the cytoplasm (**Figure 4**), whereas dead cells showed intense PI staining and absent calcein staining. The sonoporation efficiencies of Luc siNRA LPX + UTMC Luc under a range of peak negative acoustic pressures are shown in **Figure 5**. There is a significant increase in sonoporation efficiency at all acoustic pressures tested vs no treatment (*p*<0.05); however, no statistically significant further increase in sonoporation efficiency was observed above 0.25 MPa.

### *In vitro* Luc siRNA delivery with UTMC

**Figure 6:**
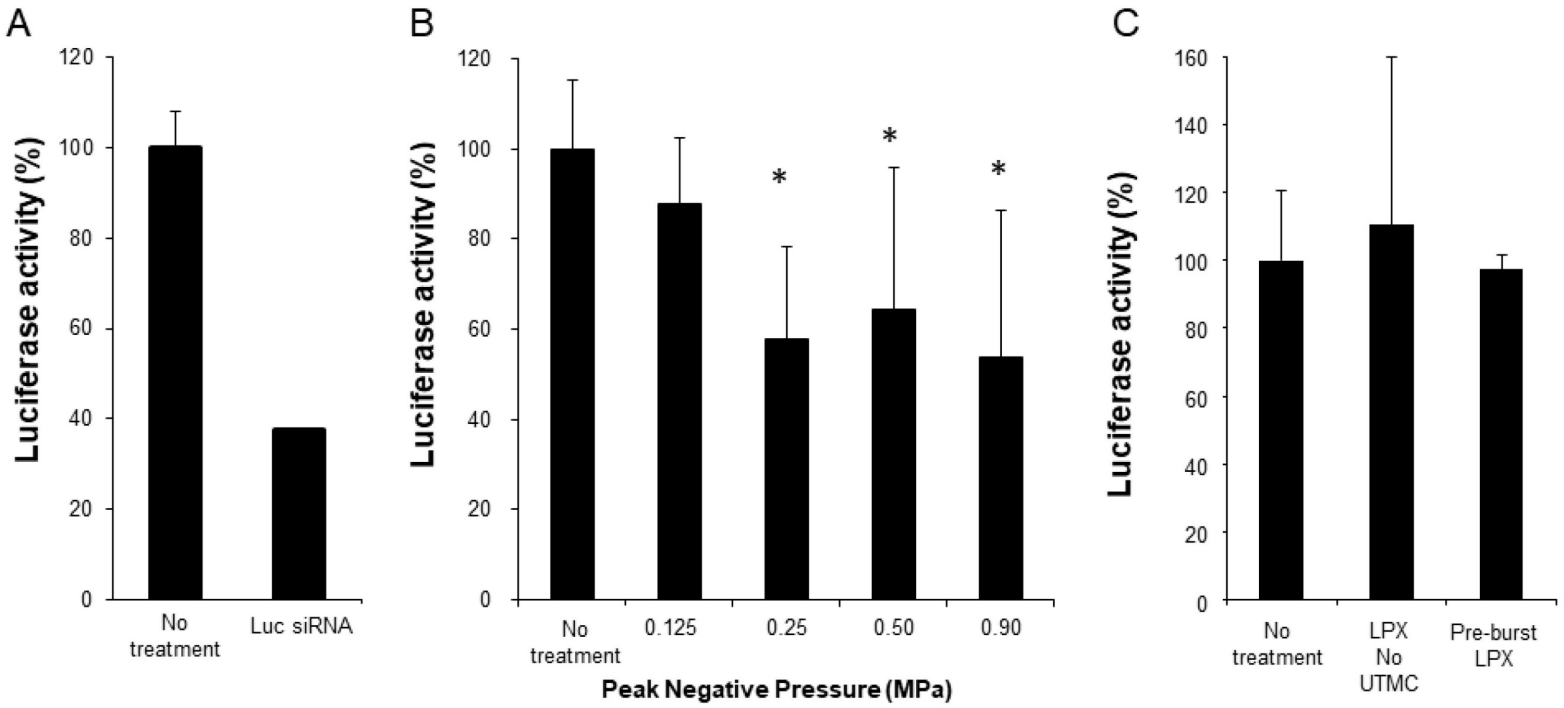
(**A**) Luciferase knockdown by Luc siRNA Lipofectamine transfection (*n*=6); (**B**) Luciferase expression decreased after treatment with Luc siRNA LPX + UTMC (*n*=6-15); (**C**) Luc siRNA LPX alone or pre-burst Luc siRNA LPX did not silence luciferase (*n*=3-6). *p<0.05 vs. no treatment control.

Functional siRNA delivery was tested using SCC-VII-Luc cells in conjunction with Luc siRNA LPX + UTMC treatment (LPX:cell ratio 60:1). As a positive control, Luc-siRNA was delivered via lipofectamine transfection, which, after 24 hours, caused ~70% knockdown of luciferase expression (**Figure 6A**). At 24 hours after Luc siRNA LPX + UTMC, there was significant luciferase knockdown (**Figure 6B**, 36%-46% vs. no treatment control, *p*<0.05) in SCC-VII-Luc cells at a range of acoustic pressures, while no statistically significant silencing effect was seen at lower pressure (0.125 MPa). Neither Luc siRNA LPX alone (no UTMC) nor pre-burst Luc siRNA LPX caused significant luciferase silencing (**Figure 6C**).

### EGFR siRNA LPX + UTMC-mediated tumor growth inhibition

**Figure 7:**
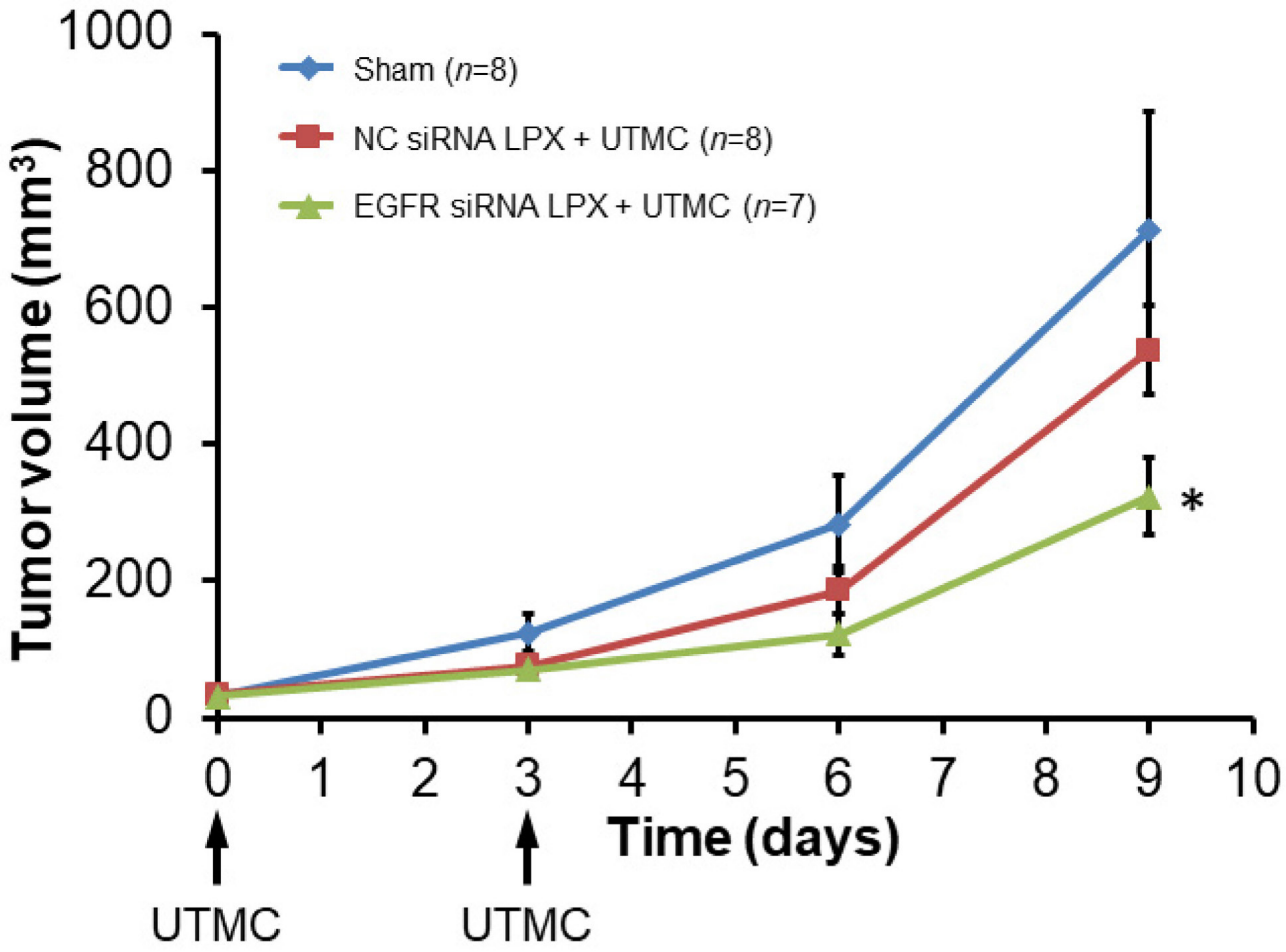
Growth of mouse SCC tumors. EGFR siRNA LPX + UTMC inhibited tumor growth compared to control treatments. * *p*<0.05 vs. NC siRNA loaded LPX + UTMC and sham groups.

Tumor growth was inhibited after treatment with EGFR siRNA LPX + UTMC. As shown in the tumor growth curves (**Figure 7**), the initial tumor volumes on day 0 for the sham, NC siRNA LPX + UTMC, and EGFR siRNA LPX + UTMC groups were similar (32.3±3.4 mm^3^, 34.4±3.3mm^3^ and 31.3±3.1mm^3^, respectively(*p*>0.05, ANOVA); on day 9, the tumor volume of mice in the EGFR siRNA LPX + UTMC group (323.8±56.0 mm^3^) was significantly smaller than the volume for sham (712.8±172.2 mm^3^) and NC siRNA LPX + UTMC (537.6±65.1mm^3^) groups (*p*<0.05 ANOVA; Student's t-test *p*<0.05 for EGFR siRNA LPX + UTMC vs. each control groups). The tumor doubling time of mice treated with EGFR siRNA LPX + UTMC (2.8±0.2 days) was significantly longer than that for sham mice (2.2±0.1 days) or mice receiving NC siRNA LPX + UTMC (2.2±0.1 days) (*p*<0.05 ANOVA; Student's *t*-test *p*<0.05 for EGFR siRNA LPX + UTMC vs. control groups).

### EGFR siRNA LPX + UTMC-mediated EGFR knockdown

**Figure 8:**
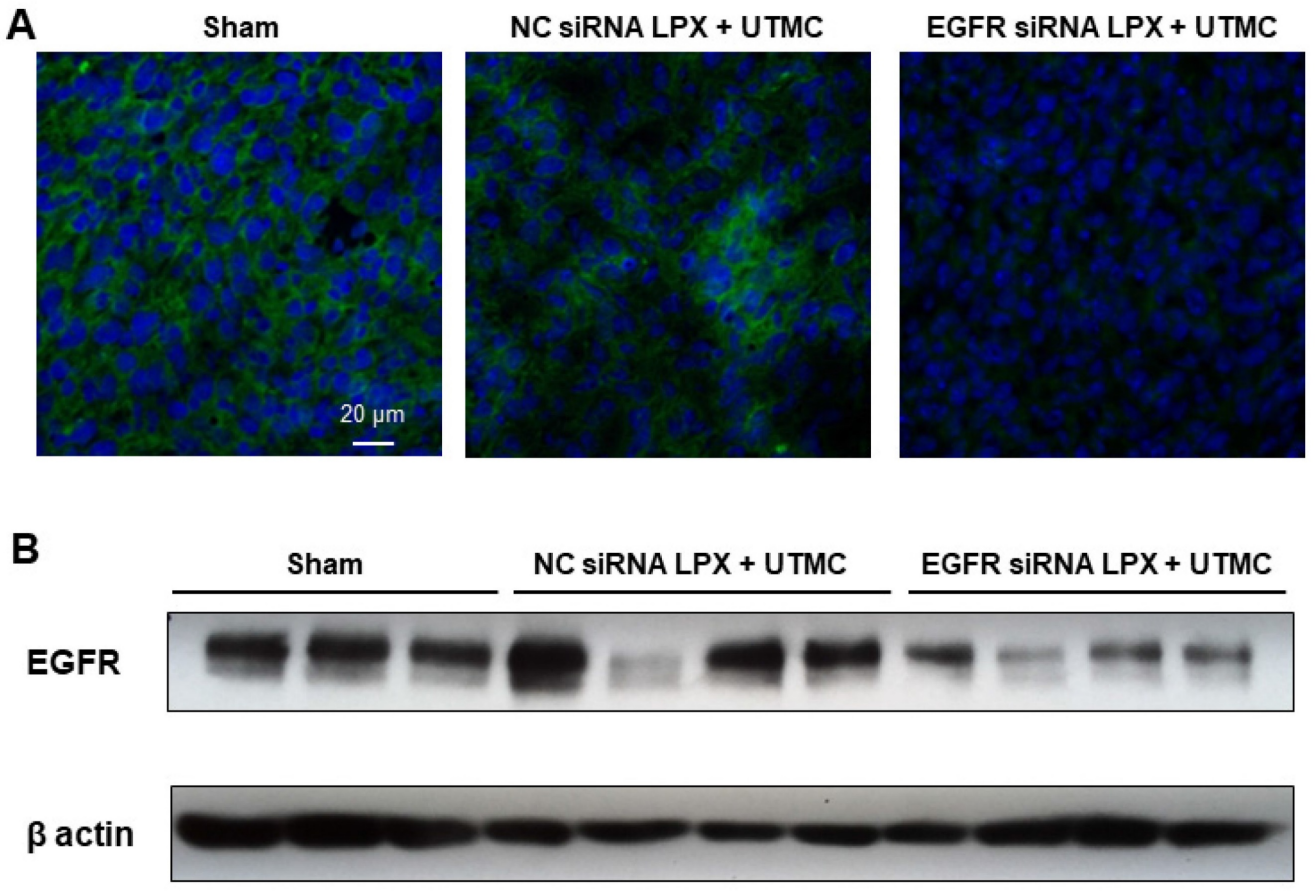
EGFR siRNA LPX + UTMC reduced tumor EGFR expression 48 h after treatment as assessed by (**A**) immunofluorescence and (**B**) Western blot.

Forty-eight hours post UTMC treatment, EGFR protein expression in tumor tissue was assessed by Western blot and immunofluorescence. Compared with NC siRNA LPX + UTMC treatment, EGFR expression was significantly reduced in tumors treated with EGFR siRNA LPX + UTMC (**Figure 8**).

### Microbubble behavior* in vivo*

**Figure 9:**
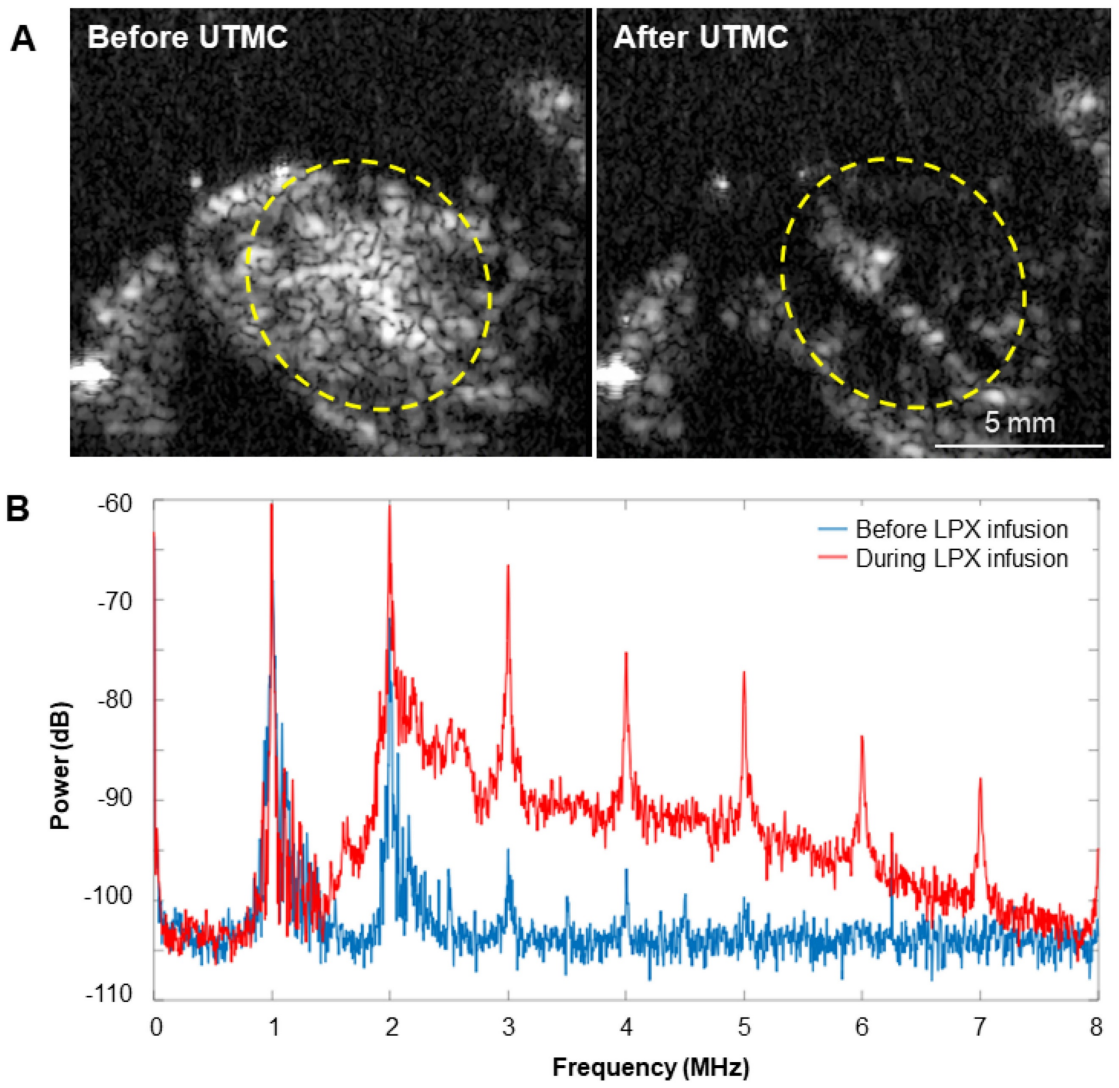
(**A**) Representative US images in contrast mode of mouse tumors immediately before and after delivery of therapy pulses indicating US-induced MB cavitation in tumors. (**B**) Power spectra from passive cavitation detection overlying the tumor before (blue) and during (red) LPX infusion and UTMC treatment. Inertial cavitation was detected during LPX infusion as evidenced by broadband noise.

Representative images of MB destruction by US and reperfusion of MBs in the tumor are shown in **Figure 9A**. During LPX + UTMC treatment, the increase in broadband signal indicated that inertial cavitation was present in the tumor (**Figure 9B**).

## Discussion

Several approaches, such as incorporating multivalent cationic lipids in the lipid shell or attaching nano-scaled complexes on the shell surface, have been adopted to improve oligonucleotide loading on MBs 23, 31, 32. Multivalent cationic lipids increase the number of charges per unit surface area, thereby enhancing oligonucleotide binding; here we have demonstrated that cationic liposome conjugated MBs (LPX) are capable of carrying siRNA. The saturation study suggests that siRNA loading capacity of MB-liposome is ~5 µg siRNA per 1×10^8^ MB, which is approximately 7-fold higher than our previously described custom-designed cationic lipid MB 19.

The combination of MBs and nanoparticles has been utilized to facilitate oligonucleotide delivery. For instance, Florinas *et al* conjugated nanocomplexes of arginine-grafted cationic bioreducible polymers and VEGF siRNA to albumin shelled perfluorocarbon (perfluoropentane or perfluorocrown ether) “microbubbles” via electrostatic interaction. In conjunction with US treatment, the “microbubble”-nanocomplex conjugate demonstrated superior target gene knockdown and tumor inhibition than the nanocomplex 31, 33. Since the boiling point of perfluoropentane and perfluorocrown ether are 29ºC and 146ºC, respectively, these constructs are droplets at room temperature, although the author called them “microbubbles.” Vandenbroucke *et al*. reported a PEG-siPlex loaded MB siRNA delivery system that was constructed by conjugating positively-charged lipoplex (siRNA: cationic liposome, N/P=20:1) to lipid MB using a biotin-avidin linker. The free, unattached lipoplex was not removed after conjugation, therefore the observed gene silencing effect after UTMC treatment may be a combinational effect of free lipoplex and the MB-lipoplex conjugate 34. In another study, plasmid loaded cationic liposomes were attached to MBs using a biotin-avidin linker. The MBs were washed after conjugation to remove free cationic liposome; the liposome-MBs were found to be capable of delivering plasmid encoded artificial miRNA to liver and reverse liver fibrosis in rats 35. In both studies the final MB constructs were positively charged. In contrast, the final construct in the present study was negatively charged since we first conjugated cationic liposomes to MBs and then used an excess amount of siRNA to saturate the cationic liposome-MB complex. This design was intentional for the following two reasons: (1) to achieve maximum loading by saturating the MB-liposome with siRNA; and (2) Saturating MBs with negatively charged nucleic acid may increase MB circulation time and decrease nonspecific adhesion 32.

To optimize US conditions for triggering efficient LPX mediated siRNA delivery, we designed a cell culture setup that can circumvent common problems encountered with conventional sonoporation setups using cell suspensions. Our setup involved culturing cells in a defined area of a sealable petri dish and inverting the culture dish filled with culture medium to allow MB flotation and MB/cell contact. This inverted setup mimics the interaction between circulating MBs and endothelial cells in the microcirculation better than previously used MB and cell suspension models 19, 36. A single element transducer with a relatively large area (25.4 mm diameter) was used to generate the US waves. The actual pressure distribution in the footprint central area (~20 mm diameter) at a 15 mm distance from the transducer surface was relatively uniform before the cell chamber was inserted, and therefore the cells were cultured in a similar circular area to allow a uniform exposure of US (**Figure 3**). Because the cell culture area was fully covered by the footprint of the transducer, transducer scanning was not required. The precise controls of exposed US to a given cell population enabled us to further investigate the effect of acoustic pressures on LPX mediated sonoporation and functional siRNA delivery.

When MBs undergo cavitation in the presence of ultrasound, the permeability of the adherent cell membrane is transiently increased. The temporal formation of pores on the cell membrane, which often occurs in a relative short time scale (from a few seconds to minutes), allows intracellular transportation of payloads 6. Direct and indirect methods have been developed to characterize sonoporation, including electron or optical microscopic observation of membrane pore opening and quantification of the intracellular delivery of membrane-impermeable molecules, such as PI or fluorescent dextran 6, 37-40. In this study, we assessed the sonoporation efficiency of LPX using PI, a membrane-impermeable dye rendering strong fluorescent signal after DNA binding in the cell nucleus. Our study demonstrated that sonoporation efficiency increases with higher acoustic pressures (**Figure 5**), in line with findings of Han *et al* 38.

We first confirmed functional siRNA delivery *in vitro* via sonication of luciferase siRNA loaded LPX in contact with luciferase expressing SCC-VII cells. Significant target gene knockdown was achieved at acoustic pressures of 0.25, 0.5 and 0.9 MPa with our pulsing scheme, while no significant knockdown was observed at the lower pressure of 0.125 MPa. In addition, cells treated with an equivalent amount of LPX without UTMC treatment or pre-burst LPX did not silence luciferase (**Figure 6**), indicating LPX mediated siRNA delivery was US-dependent. This US-dependent behavior is advantageous for *in vivo* applications, as ideally, siRNA should only be delivered to the target tissues, such as tumor.

Unlike other nanoparticle-based gene delivery vehicles, attributes of the surrounding environments, such as viscosity, can have a significant impact on US-stimulated MB dynamics, which in turn can further influence drug/gene delivery capacity. The dynamic behavior of phospholipid encapsulated MBs under different viscosity conditions which are present in saline (1 cP) or in vessels (4 cP) has been systemically investigated. Our previous reports showed both MB oscillation and cavitation threshold varies from under these two viscosity conditions 30, 41. Our *in vitro* knockdown experiment, performed in cell culture medium (viscosity ~1cp), indicates that 0.25 MPa is a threshold pressure for functional siRNA delivery. To compensate for the effect of blood viscosity on MB activity, we chose a higher acoustic pressure (0.5 MPa) for the *in vivo* assessment of LPX mediated siRNA delivery in our mouse tumor model. Passive cavitation detection *in vivo* demonstrated that inertial cavitation was present in the tumor during EGFR siRNA LPX + UTMC treatment (**Figure 7**). *In vivo* tests showed that 0.5 MPa was sufficient to destroy circulating LPX in tumor vasculature (**Figure 7**).

EGFR was the disease target of our *in vivo* study. Overexpression of EGFR occurs in solid tumors such as breast, lung, and head and neck cancers 19. Clinically, blocking EGFR with monoclonal antibody or tyrosine kinase inhibitor has conferred some, but limited clinical benefits as monotherapy, and acquired resistance to ant-EGFR agents has defined a need for alternative therapies 42, 43. As such, silencing of EGFR expression using RNA interference is a promising anti-EGFR therapy 44-47.

Previously, we showed our custom-designed cationic lipid MB could deliver EGFR siRNA to SCC-VII tumor cells using a diagnostic US pulse available on a clinical system, trigger target gene silencing, and induce tumor growth inhibition 19. In the current study, we demonstrated that a new LPX formulation loading more siRNA than previously possible, combined with a well-defined acoustic regime with a single element transducer, could functionally deliver EGFR siRNA to tumor cells, resulting in a decrease in tumor burden and a reduction in EGFR expression compared to negative controls.

One limitation for the current LPX platform is that biotin-streptavidin linkage was used to attach liposomes to MBs. This linker was chosen because it is simple and convenient for proof-of-concept studies. Streptavidin is potentially immunogenic because it is not an endogenous protein in humans. However, this issue could be solved by switching to other covalent linkers, such as a disulfide bond or thiol-ether bond. Also, while we were able to load more siRNA per MB with our LPX platform, future studies will need to test its therapeutic efficacy in comparison to our standard cationic lipid MB formulation 19, 48. Due to the higher siRNA loading capacity per LPX, we would anticipate comparable anti-tumor efficacy at a lower LPX concentration compared to the cationic lipid MB preparation, or perhaps higher therapeutic efficacy of LPX formulation for a given comparable LPX and cationic lipid MB concentration. Further, as shown in Figure 7, whereas UTMC delivery of siRNA on Days 0 and 3 inhibited tumor growth in the initial week, in the absence of further treatment, tumor size increased in the ensuing days, consistent with loss of siRNA inhibition over time. Our data suggest that, as with cyclic chemotherapy or radiotherapy, repeated UTMC treatments will be required to achieve a fully therapeutic effect if given alone. Alternatively, UTMC can be combined with chemotherapy or radiotherapy for possible augmented or synergistic effects, for which further studies are required.

Finally, another requirement for clinical translation will be to further understand the fate of the LPX construct. We did not perform biodistribution studies in these proof-of-concept experiments. Having established the therapeutic potential of the LPX platform, the next steps would include studies to determine the pharmacokinetics and pharmacodynamics of our novel construct.

## Conclusion

Our new formulation of siRNA-loaded LPXs, combined with pulsed US, achieved functional delivery of siRNA to SCC-VII tumor cells *in vitro*. When loaded with EGFR-siRNA, LPX + UTMC suppressed EGFR expression and inhibited tumor growth *in vivo*, suggesting that this platform holds promise for non-invasive, image-guided targeted delivery of therapeutic siRNA for cancer treatment. Given that LPX loads nearly 1 order of magnitude more siRNA compared to our standard cationic lipid MB formulation, it promises to achieve tumor suppression at lower MB doses, which should facilitate clinical translation.

## Abbreviations

EGFR: epidermal growth factor receptor
EGFR siRNA LPX: microbubble liposome complexes loaded with EGFR siRNA
FBS: fetal bovine serum
siRNA: small interfering RNA
Luc siRNA: siRNA against firefly luciferase
Luc siRNA LPX: microbubble liposome complexes loaded with luciferase siRNA
LPX: microbubble-liposome complexes
MB: microbubble
NC siRNA LPX: microbubble liposome complexes loaded with negative control siRNA
PI: propidium iodine
SCC-VII: Murine squamous cell carcinoma
SCC-VII-Luc: luciferase expressing SCC-VII cells
siRNA: small interfering RNA
US: ultrasound
UTMC: ultrasound-targeted microbubble cavitation

## References

[1] Ptasznik A, Nakata Y, Kalota A, Emerson SG, Gewirtz AM (2004). Short interfering RNA (siRNA) targeting the Lyn kinase induces apoptosis in primary, and drug-resistant, BCR-ABL1(+) leukemia cells. Nat Med.

[2] Sato Y, Murase K, Kato J, Kobune M, Sato T, Kawano Y (2008). Resolution of liver cirrhosis using vitamin A-coupled liposomes to deliver siRNA against a collagen-specific chaperone. Nat Biotechnol.

[3] Schiffelers RM, Ansari A, Xu J, Zhou Q, Tang Q, Storm G (2004). Cancer siRNA therapy by tumor selective delivery with ligand-targeted sterically stabilized nanoparticle. Nucleic Acids Res.

[4] Whitehead KA, Langer R, Anderson DG (2009). Knocking down barriers: advances in siRNA delivery. Nat Rev Drug Discov.

[5] Sirsi S, Borden M (2009). Microbubble Compositions, Properties and Biomedical Applications. Bubble Sci Eng Technol.

[6] Helfield B, Chen X, Watkins SC, Villanueva FS (2016). Biophysical insight into mechanisms of sonoporation. Proc Natl Acad Sci U S A.

[7] Ramasamy T, Chen X, Qin B, Johnson DE, Grandis JR, Villanueva FS (2020). STAT3 decoy oligonucleotide-carrying microbubbles with pulsed ultrasound for enhanced therapeutic effect in head and neck tumors. Plos One.

[8] Kopechek JA, McTiernan CF, Chen X, Zhu JH, Mburu M, Feroze R (2019). Ultrasound and Microbubble-targeted Delivery of a microRNA Inhibitor to the Heart Suppresses Cardiac Hypertrophy and Preserves Cardiac Function. Theranostics.

[9] Kopechek JA, Carson AR, McTiernan CF, Chen X, Klein EC, Villanueva FS (2016). Cardiac Gene Expression Knockdown Using Small Inhibitory RNA-Loaded Microbubbles and Ultrasound. Plos One.

[10] Kopechek JA, Carson AR, McTiernan CF, Chen X, Hasjim B, Lavery L (2015). Ultrasound Targeted Microbubble Destruction-Mediated Delivery of a Transcription Factor Decoy Inhibits STAT3 Signaling and Tumor Growth. Theranostics.

[11] Bekeredjian R, Kroll RD, Fein E, Tinkov S, Coester C, Winter G (2007). Ultrasound targeted microbubble destruction increases capillary permeability in hepatomas. Ultrasound Med Biol.

[12] Mullin LB, Phillips LC, Dayton PA Nanoparticle delivery enhancement with acoustically activated microbubbles. IEEE Trans Ultrason Ferroelectr Freq Control. 60: 65-77.

[13] Price RJ, Skyba DM, Kaul S, Skalak TC (1998). Delivery of colloidal particles and red blood cells to tissue through microvessel ruptures created by targeted microbubble destruction with ultrasound. Circulation.

[14] Zhou Y, Yang K, Cui J, Ye JY, Deng CX Controlled permeation of cell membrane by single bubble acoustic cavitation. J Control Release. 157: 103-11.

[15] Kinoshita M, Hynynen K (2005). A novel method for the intracellular delivery of siRNA using microbubble-enhanced focused ultrasound. Biochem Biophys Res Commun.

[16] Christiansen JP, French BA, Klibanov AL, Kaul S, Lindner JR (2003). Targeted tissue transfection with ultrasound destruction of plasmid-bearing cationic microbubbles. Ultrasound Med Biol.

[17] Panje CM, Wang DS, Pysz MA, Paulmurugan R, Ren Y, Tranquart F Ultrasound-mediated gene delivery with cationic versus neutral microbubbles: effect of DNA and microbubble dose on in vivo transfection efficiency. Theranostics. 2: 1078-91.

[18] Wang DS, Panje C, Pysz MA, Paulmurugan R, Rosenberg J, Gambhir SS Cationic versus neutral microbubbles for ultrasound-mediated gene delivery in cancer. Radiology. 264: 721-32.

[19] Carson AR, McTiernan CF, Lavery L, Grata M, Leng X, Wang J (2012). Ultrasound-targeted microbubble destruction to deliver siRNA cancer therapy. Cancer Res.

[20] Sun L, Huang CW, Wu J, Chen KJ, Li SH, Weisel RD The use of cationic microbubbles to improve ultrasound-targeted gene delivery to the ischemic myocardium. Biomaterials. 34: 2107-16.

[21] Chen S, Ding JH, Bekeredjian R, Yang BZ, Shohet RV, Johnston SA (2006). Efficient gene delivery to pancreatic islets with ultrasonic microbubble destruction technology. Proc Natl Acad Sci U S A.

[22] Navarro-Becerra JA, Borden MA (2023). Targeted Microbubbles for Drug, Gene, and Cell Delivery in Therapy and Immunotherapy. Pharmaceutics.

[23] Jin QF, Wang ZY, Yan F, Deng ZT, Ni F, Wu JR (2013). A Novel Cationic Microbubble Coated with Stearic Acid-Modified Polyethylenimine to Enhance DNA Loading and Gene Delivery by Ultrasound. Plos One.

[24] Jun J, Shang-Yi J, Xia H, Wen-Ping L (2011). Preparation of ultrasound microbubbles crosslinked to albumin nanoparticles packaged with tissue-type plasminogen activator gene plasmid and method of in vivo transfection. J Exp Pharmacol.

[25] Garg S, Thomas AA, Borden MA (2013). The effect of lipid monolayer in-plane rigidity on in vivo microbubble circulation persistence. Biomaterials.

[26] Wu SY, Chen CC, Tung YS, Olumolade OO, Konofagou EE (2015). Effects of the microbubble shell physicochemical properties on ultrasound-mediated drug delivery to the brain. J Control Release.

[27] Kotopoulis S, Lam C, Haugse R, Snipstad S, Murvold E, Jouleh T (2022). Formulation and characterisation of drug-loaded antibubbles for image-guided and ultrasound-triggered drug delivery. Ultrason Sonochem.

[28] Lentacker I, De Smedt SC, Sanders NN (2009). Drug loaded microbubble design for ultrasound triggered delivery. Soft Matter.

[29] Dewitte H, Vanderperren K, Haers H, Stock E, Duchateau L, Hesta M (2015). Theranostic mRNA-loaded microbubbles in the lymphatics of dogs: implications for drug delivery. Theranostics.

[30] Helfield B, Black JJ, Qin B, Pacella J, Chen X, Villanueva FS Fluid Viscosity Affects the Fragmentation and Inertial Cavitation Threshold of Lipid-Encapsulated Microbubbles. Ultrasound Med Biol. 42: 782-94.

[31] Florinas S, Nam HY, Kim SW (2013). Enhanced siRNA delivery using a combination of an arginine-grafted bioreducible polymer, ultrasound, and microbubbles in cancer cells. Mol Pharm.

[32] Sirsi SR, Hernandez SL, Zielinski L, Blomback H, Koubaa A, Synder M Polyplex-microbubble hybrids for ultrasound-guided plasmid DNA delivery to solid tumors. J Control Release. 157: 224-34.

[33] Florinas S, Kim J, Nam K, Janat-Amsbury MM, Kim SW (2014). Ultrasound-assisted siRNA delivery via arginine-grafted bioreducible polymer and microbubbles targeting VEGF for ovarian cancer treatment. J Control Release.

[34] Vandenbroucke RE, Lentacker I, Demeester J, De Smedt SC, Sanders NN (2008). Ultrasound assisted siRNA delivery using PEG-siPlex loaded microbubbles. J Control Release.

[35] Yang D, Gao YH, Tan KB, Zuo ZX, Yang WX, Hua X Inhibition of hepatic fibrosis with artificial microRNA using ultrasound and cationic liposome-bearing microbubbles. Gene Ther. 20: 1140-8.

[36] Yu FT, Chen X, Wang J, Qin B, Villanueva FS (2016). Low intensity ultrasound mediated liposomal doxorubicin delivery using polymer microbubbles. Molecular pharmaceutics.

[37] Fan Z, Chen D, Deng CX (2013). Improving ultrasound gene transfection efficiency by controlling ultrasound excitation of microbubbles. J Control Release.

[38] Han YW, Ikegami A, Chung P, Zhang L, Deng CX (2007). Sonoporation is an efficient tool for intracellular fluorescent dextran delivery and one-step double-crossover mutant construction in Fusobacterium nucleatum. Appl Environ Microbiol.

[39] Kooiman K, Foppen-Harteveld M, de Jong N (2010). Ultrasound-mediated targeted microbubble sonoporation of endothelial cells. J Control Release.

[40] Meijering BD, Juffermans LJ, van Wamel A, Henning RH, Zuhorn IS, Emmer M (2009). Ultrasound and microbubble-targeted delivery of macromolecules is regulated by induction of endocytosis and pore formation. Circ Res.

[41] Helfield B, Chen X, Qin B, Villanueva FS Individual lipid encapsulated microbubble radial oscillations: Effects of fluid viscosity. J Acoust Soc Am. 139: 204.

[42] Normanno N, De Luca A, Bianco C, Strizzi L, Mancino M, Maiello MR (2006). Epidermal growth factor receptor (EGFR) signaling in cancer. Gene.

[43] Vokes EE, Chu E (2006). Anti-EGFR therapies: clinical experience in colorectal, lung, and head and neck cancers. Oncology.

[44] Chen G, Kronenberger P, Teugels E, Umelo IA, De Greve J (2012). Targeting the epidermal growth factor receptor in non-small cell lung cancer cells: the effect of combining RNA interference with tyrosine kinase inhibitors or cetuximab. BMC medicine.

[45] Li SD, Chen YC, Hackett MJ, Huang L (2008). Tumor-targeted delivery of siRNA by self-assembled nanoparticles. Molecular therapy: the journal of the American Society of Gene Therapy.

[46] Nozawa H, Tadakuma T, Ono T, Sato M, Hiroi S, Masumoto K (2006). Small interfering RNA targeting epidermal growth factor receptor enhances chemosensitivity to cisplatin, 5-fluorouracil and docetaxel in head and neck squamous cell carcinoma. Cancer science.

[47] Satpathy M, Mezencev R, Wang L, McDonald JF (2016). Targeted in vivo delivery of EGFR siRNA inhibits ovarian cancer growth and enhances drug sensitivity. Scientific reports.

[48] Carson AR, McTiernan CF, Lavery L, Hodnick A, Grata M, Leng X (2011). Gene therapy of carcinoma using ultrasound-targeted microbubble destruction. Ultrasound Med Biol.

